# On-station performance evaluation of improved tropically adapted chicken breeds for smallholder poultry production systems in Nigeria

**DOI:** 10.1007/s11250-019-02158-9

**Published:** 2019-12-08

**Authors:** O. Bamidele, E. B. Sonaiya, O. A. Adebambo, T. Dessie

**Affiliations:** 1 African Chicken Genetic Gains, c/o Department of Animal Sciences, Obafemi Awolowo University, Ile, Ife, Nigeria; 2 Department of Animal Genetics and Breeding, Federal University of Agriculture, Abeokuta, Nigeria; 3 International Livestock Research Institute, Addis Ababa, Ethiopia

**Keywords:** Chickens, Smallholder poultry, On-station, Dual-purpose, Breeds

## Abstract

**Background:**

Availability of appropriate genetics is important for the development of smallholder poultry (SHP). The biological potential of improved dual purpose chicken germplasms was evaluated in Nigeria.

**Methods:**

A total of six breeds (*Fulani*, *FUNAAB Alpha*, *Kuroiler*, *Noiler*, *Sasso*, and *Shika-Brown*) were tested on-station, in deep litter houses at two test centres (Public and private facility) for 504 days. Birds were fed ad libitum for the first 140 days after which restricted feeding was practised.

**Result:**

Lowest and highest hatchability of eggs set was 55% (*FUNAAB Alpha*) and 89% (*Sasso*), respectively. At 140 days, male live weights were 200%–300 % higher than the local chickens (975 g) except *Shika-Brown* (152%) and *Fulani* (135%). Lowest (*p* < 0.05) age at first egg was 119 days for *Shika-Brown* and 120 days for *FUNAAB Alpha* and *Kuroiler.* Highest hen-housed egg production was 192 for *Shika-Brown*, and feed intake per dozen eggs was lowest (*p* < 0.05) for *Shika-Brown* (2.9 kg) and *FUNAAB Alpha* (2.9 kg). Mortality rate of the locally sourced breeds (*Fulani*, *FUNAAB Alpha*, *Noiler*, *and Shika-Brown*) was significantly lower (*p* < 0.05) than the foreign-sourced breeds (*Kuroiler*, *Sasso*) during brooding, growing and laying.

**Conclusion:**

Results from this study identified *FUNAAB Alpha* and *Noiler* as being more suitable for dual-purpose functions (egg and meat), while *Sasso* and *Kuroiler* (meat) and *Shika-Brown* (egg) were observed to be better suited for single purpose functions. These findings could guide the introduction of smallholder poultry-specific hybrid germplasms for the development of the smallholder poultry production systems in Nigeria.

## Introduction

Smallholder poultry (SHP) is defined as poultry keeping by households using family labour, locally available feed resources obtained largely through scavenging by a flock of less than 100 birds, of unimproved or improved breed (Sonaiya, 1990). The characteristics and importance of SHP are fundamentally similar in rural and peri-urban farming communities irrespective of the sociocultural factors and agroecological conditions (FAO, 2010). The low productivity of the SHP production systems (as characterized by high mortality, low egg numbers, and poor live performance) has resulted in the demand by SHP producers for “better and bigger” birds in Nigeria (ACGG Nigeria baseline data, 2016). Such “better” birds are best provided not by commercial/industrial poultry germplasms but by SHP-specific hybrid germplasms (SHP-SHG) which are improved dual-purpose birds (meat and egg production) that incorporate genes for higher productivity and performance into the hardiness of locally adapted chickens.

The introduction of SHP-SHG has been successfully tested on-farm, under scavenging management systems (i.e. Backyard poultry), in Bangladesh for the Sonali chickens (FAO, 2015), and in Uganda with the *Kuroiler* (Galukande et al., 2016; Sharma et al., 2015). However, under the African Chicken Genetic Gains project in Nigeria, the testing of six SHP-SHG (*Fulani*, *FUNAAB Alpha*, *Noiler*, *Kuroiler*, *Sasso*, and *Shika*-*Brown*) was conducted both on-farm, as a farmer-led experimentation, and on-station (https://africacgg.net). The on-station research condition (controlled environment) was adopted as an evidenced-based approach of investigating the biological potential of SHP-SHG under intensive management systems, with optimum feeds and feeding, housing, biosecurity, and health measures. Hence, the objective of this study was to evaluate the growth performance, egg production, and mortality rate of the selected SHP-SHG, under on-station conditions in Nigeria.

## Material and methods

### Study locations

The on-station test was conducted at a private (Fol-Hope Farms, Ibadan, Oyo State) and public (Federal University of Agriculture, Abeokuta, Ogun State – FUNAAB) facility located within the southern Guinea savanna, and dry lowland rainforest of Nigeria, respectively. The birds were tested in isolated poultry houses sited away from other birds at the test centres. The coordinates of the two test centres, within the respective agroecologies, were: latitude 7°N 20' 25", longitude 3°E 58' 14" and altitude 4.5 m (Fol-Hope station), and latitude 7°N 13'28", longitude 3°E 26' 3"and altitude 133.8 m (FUNAAB station). Testing of the breeds commenced in May 2016, and lasted for 72 weeks. During the test period, the minimum and maximum temperature (°C) at Fol-Hope was 17.8 °C and 36.4 °C, while at FUNAAB it was 18.3 °C and 39.7 °C. Average daily temperature (°C) ranged from 30.9 °C (Day)–23.1 °C (Night) at Fol-Hope, and 33.1 °C (Day)–26.4 °C (Night) at FUNAAB. The average annual rainfall (mm) and relative humidity (%), at Fol-Hope and FUNAAB test stations, were 1345.5 mm and 79.5% and 1030.1 mm and 84.4%, respectively (NiMET, 2019).

### Experimental birds and management

Hatchable eggs of the locally sourced breeds (*Fulani*, *FUNAAB Alpha*, *Noiler* and *Shika-Brown*) were purchased from the respective breeder farms (1st Agro Limited, Abeokuta, Ogun State; PEARL Poultry Unit, FUNAAB, Ogun State; and Amo Farms Sieberer Hatchery Limited, Awe, Oyo State, and Fol-Hope Farms Limited, Ibadan, Oyo State) within the country. International veterinary certificates were obtained from the Federal Department of Veterinary and Pest Control Services, Federal Ministry of Agriculture and Rural Development (FMARD), Abuja, Federal Capital Territory for the importation of hatchable eggs, of *Kuroiler* from Chick Masters Limited, Mukono, Uganda (FDL/BHE/12/16), and *Sasso*, from Silverlands Tanzania Limited, Iringa, Tanzania (FDL/BHE/11/16). All the chicks were hatched on-station, and a total of 1,939-day-old chicks of both locally sourced breeds and foreign-sourced breeds were brooded from 0 to 42 days (d). The birds were sexed at 42 days, and grown separately until 140 days for males and 504 days for females. The population density was 10 chicks/m^2^, 7 birds/m^2^, and 5 birds/m^2^ during brooding, growing, and laying phases, respectively. Commercial feed (Chick mash: 2,993 kcal ME/kg, 22.3% CP; Grower mash: 3,013 kcal ME/kg, 17% CP) and water were available ad libitum during brooding and growing phases. During the laying phase (Layer mash: 2,500 kcal ME/kg, 16.5% CP, 3.6% Ca), hens were restricted to a maximum of 120 g feed/hen/day. Standard biosecurity measures and vaccination schedules were observed at the test centres. This study was approved by the International Livestock Research Institute Institutional Animal Care and Use Committee (ILRI IACUC) with reference number: IACUC-RC2016.26.

### Data collection and analysis

Fertility and hatchability percentages were determined from hatchery performance record taken at day 1 (eggs set), day 18 (candling), and day 21 (hatching)**.** Data on feed intake, egg production, and mortality were taken daily. Body weights were measured every 14 days. For males, bodyweights were taken until 140 days when final live weights were obtained for meat production. For females, all measurements were taken up till 504 days, with particular interest in egg production. The temperature humidity index (THI), as an indicator of heat stress, was determined and categorised (normal: < 27.8, moderate: 27.8–28.8, severe: 28.9–29.9, and very severe: ≥ 30.0) as described by Lallo et al. (2018). This study was conducted independently in each of the test stations as a randomised complete block design (RCBD) of 4 replicates (pens) per breed. The dimension of the pens was 1.8 m × 1.3 m. Growth rate, feed conversion ratio (FCR), hen-housed egg production (HHEP), hen-day egg production (HDEP), and mortality rate were analysed using a two-way analysis of variance, with breed and location as factors (SAS university edition). Differences between means were separated using Duncan’s multiple range test.

## Results

Table 1 shows the hatchery performance. Hatchability of the locally sourced breeds was 68.2%, while that of the foreign-sourced breeds was 70%. Hatchability of eggs set (HES) ranged from 55% (*Fulani* and *FUNAAB Alpha*) to 84% (*Noiler*). Hatchability of fertile eggs (HFE) was 80% and above, in all the breeds, except *Fulani* (72%).

**Table 1:** Hatchability of eggs of the six breeds

Breeds	Eggs set*	Number fertile	Hatchability % of eggs set (HES)	Hatchability % of fertile eggs (HFE)
Fulani	8,187	6,275	55	72
FUNAAB Alpha	13,688	9,153	55	82
Kuroiler	24,480	20,521	67	80
Noiler	11,905	11,190	84	89
Sasso	93,992	79,629	71	84
Shika-Brown	23,157	20,911	77	85

*Total number of eggs set for both on-station and on-farm test

Table 2 shows the live weight performance of the breeds. Bodyweight gain and FCR of the breeds differed (*p* < 0.05) significantly during brooding, growing, and laying. *Noiler* had the lowest FCR at brooding (2.2), while FCR for *Kuroiler* (7.5) and *Sasso* (7.6) were lowest during growing for males. For growing females, *Kuroiler* (9.8), *Noiler* (9.7), and *Sasso* (9.8) had the lowest FCR but were not significantly different. Male bodyweight for *Sasso* (2,962.1 g) and *Kuroiler* (2,894.2 g) was higher (*p* < 0.05) than *Noiler* (2,599.0 g), while *FUNAAB Alpha* (2,097.0 g) differed (*p* < 0.05) from *Shika-Brown* (1688.4 g) and *Fulani* (1321.0 g). At 504 days, *Sasso* had the highest live weight (2634.4 g) but did not differ (*p* < 0.05) from Kuroiler (2422.0 g), and Noiler (2355.1 g). Also, FUNAAB Alpha (2132.7 g) was similar (*p* < 0.05) to *Kuroiler*, *Noiler*, *Sasso*, and *Shika-Brown* (2045.7 g) but higher than *Fulani* (1740.2 g). The genotype by environment interaction on final bodyweight and FCR across the rearing phases was not significant (*p* < 0.05), except for the FCR during growing for females.

**Table 2:** Body weight and feed performance (LSM ± SEM) of the six breeds tested on-station

Parameters	Breed						Station	
	Fulani	FUNAAB Alpha	Kuroiler	Noiler	Sasso	Shika-Brown	Fol-Hope	FUNAAB
Brooding	Both sexes							
	(70)	(353)	(408)	(310)	(408)	(400)	(983)	(966)
Day-old BW, g	24.8 ± 0.9^d^	31.4 ± 0.9^b^	40.4 ± 0.6^a^	37.1 ± 0.7 ^ab^	39.0 ± 0.9^a^	28.0 ± 0.3^c^	33.5 ± 0.3^a^	33.2 ± 0.5^a^
42-day BW, g	202.1 ± 14.8^d^	378.8 ± 11.7^c^	598.0 ± 22.3^b^	744.1 ± 10.6^a^	494.6 ± 8.9^b^	279.0 ± 4.5^cd^	454.8 ± 2.2^a^	442.1 ± 7.1^a^
42-day FI, g/bird	776.8 ± 9.7^e^	1092.6 ± 9.1^d^	1734.5 ± 18.4^a^	1575.3 ± 9.2^b^	1385.1 ± 21.1^c^	1046.8 ± 8.0^d^	1250.2 ± 15.9^a^	1262.8 ± 11.6^a^
FCR	4.4 ± 2.1^d^	3.2 ± 1.7^b^	3.1 ± 1.8^b^	2.2 ± 0.5^a^	3.0 ± 1.2^b^	4.2 ± 1.4^c^	3.0 ± 1.3^a^	3.1 ± 0.7^a^
Growing	Male birds							
	(20)	(113)	(130)	(148)	(190)	(194)	(380)	(415)
56-day BW, g	365.5 ± 25.8^e^	684.5 ± 18.3^c^	969.2 ± 22.3^b^	1179.2 ± 19.8^a^	919.9 ± 15.4^b^	501.5 ± 8.9^d^	866.5 ± 20.6^a^	681.3 ± 19.4^b^
140-day BW, g	1321.0 ± 84.7^e^	2097.0 ± 62.6^c^	2894.2 ± 51.0^a^	2599.0 ± 51.0^b^	2962.1 ± 48.9^a^	1688.4 ± 27.3^d^	2257.6 ± 18.0^a^	2264.8 ± 14.5^a^
98-day FI, g/bird	9705.3 ± 22.1^c^	15621.7 ± 31.4^a^	14442.4 ± 28.3^a^	12395.1 ± 40.8^b^	15575.5 ± 36.0^a^	12451.6 ± 30.1^b^	12849.7 ± 27.1^a^	13880.5 ± 32.5^a^
FCR	10.2 ± 3.1^c^	11.0 ± 2.2^d^	7.5 ± 1.7^a^	8.7 ± 0.9^b^	7.6 ± 1.6^a^	10.5 ± 2.3^c^	9.2 ± 2.5^a^	8.7 ± 3.1^a^
Growing	Female birds							
	(46)	(180)	(141)	(151)	(181)	(198)	(468)	(429)
56-day BW, g	325.0 ± 10.5^d^	594.0 ± 15.8^c^	833.2 ± 25.5^b^	975.5 ± 15.5^a^	854.2 ± 11.7^b^	472.0 ± 6.4^cd^	676.8 ± 12.3^a^	677.2 ± 10.8^a^
140-day BW, g	1015.6 ± 47.2^c^	1635.0 ± 54.5^b^	2411.7 ± 35.6^a^	2045.3 ± 41.7^a^	2334.7 ± 19.0^a^	1334.6 ± 25.2^bc^	1759.1 ± 39.7^a^	1821.7 ± 23.6^a^
98-day FI, g/bird	9133.1 ± 28.2^bc^	15166.7 ± 42.7^a^	15450.4 ± 36.7^a^	10307.0 ± 40.0^b^	14495.3 ± 78.5^a^	10962.1 ± 19.3^b^	13373.4 ± 51.4^a^	11798.5 ± 47.1^b^
FCR	13.3 ± 3.1^b^	14.6 ± 4.6^c^	9.8 ± 3.7^a^	9.7 ± 2.1^a^	9.8 ± 3.4^a^	12.8 ± 2.7^b^	11.9 ± 3.1^b^	10.4 ± 3.8^a^
Laying	(31)	(162)	(139)	(141)	(179)	(194)	(443)	(403)
504-day BW, g	1740.2 ± 48.2^c^	2132.7 ± 22.6^ab^	2422.0 ± 30.1^a^	2355.1 ± 25.9^a^	2634.4 ± 34.0^a^	2045.7 ± 18.6^b^	2294.3 ± 19.0^a^	2149.2 ± 25.7^a^

*BW* bodyweight, *FI* feed intake, *FCR* feed conversion ratio, *M* male, *F* female, ( ) figures in parenthesis are number of observations at the start of the phase

^a-e^Means with different superscripts on the same row were significantly different at *p* < 0.05, *LSM* least square mean, *SEM* standard error of the mean

**Fig. 1:**
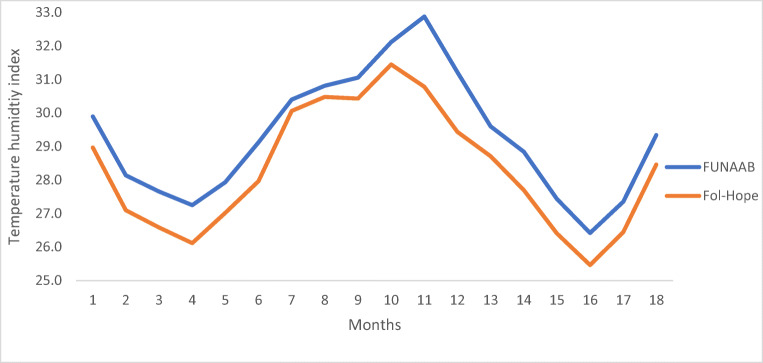
Average temperature humidity index (THI) during the test period at the on-station centres

Tables 3 and 4 show the laying performance. The genotype by environment interaction was significant (*p* < 0.05) for all the parameters, except egg weight (Table 3). The mean ages at first egg, egg weight, and HHEP in 364 days were 125 days, 52 g, and 141 days, respectively. *Shika-Brown* had the lowest (*p* < 0.05) age at first egg (119 days). *Noiler* and *Shika-Brown* had the highest (*p* < 0.05) egg weight (56 g) which was not significantly different from that of *Kuroiler* and *Sasso*. The peak HDEPs for *Fulani* (56.1%), *FUNAAB Alpha* (63.9%), *Kuroiler* (65.4%), *Noiler* (55.6%), *Sasso* (36.5%), and *Shika-Brown* (68.1%) were at 26, 26, 29, 41, 30, and 35 weeks, respectively. Feed intake per dozen eggs was lowest (*p* < 0.05) for *Shika-Brown* (2.9 kg) and *FUNAAB Alpha* (2.9 kg). Table 4 shows that age at first egg ranged from 116 (Shika-Brown) to 143 (Noiler) at Fol-Hope, and from 116 (Noiler) to 123 (Sasso) at FUNAAB. *Shika-Brown* and *FUNAAB Alpha* both had the highest (*p* < 0.05) HHEP at the two test stations. Feed per dozen eggs was lowest for *FUNAAB Alpha* (2.4) and *Shika-Brown* (2.6) at Fol-Hope, and *Shika-Brown* (3.2), *Noiler* (3.4), and *FUNAAB Alpha* (3.6) at FUNAAB. At Fol-Hope, the highest peak HDEP was observed at weeks 26 (75.3%, *FUNAAB Alpha*) and 35 (70.7%, *Shika-Brown*), while at FUNAAB, it was at weeks 25 (67.9%, *Fulani*) and 33 (64.9%, *Shika-Brown*). Figure 1 shows the average monthly THI for the entire test period, with an average of 28.8 ± 1.8 (Fol-Hope), and 29.4 ± 1.9 (FUNAAB) during the 13-month-laying phase. There was a total of 8 (FUNAAB) and 11 (Fol-Hope) months of normal–moderate (27.8–28.8), and 7 (Fol-Hope) and 10 (FUNAAB) months of severe–very severe (28.9– ≥ 30) THI values.

**Table 3:** Genotype by environment interaction (LSM ± SEM) on laying performance at the test stations

Parameters	Breed						Station	
	Fulani	FUNAAB Alpha	Kuroiler	Noiler	Sasso	Shika-Brown	Fol-Hope	FUNAAB
Age at first egg, d	126 ± 1.1^c^	120 ± 0.9^b^	120 ± 1.3^b^	130 ± 1.8^d^	133 ± 0.6^d^	119 ± 0.3^a^	128 ± 0.9^b^	120 ± 0.8^a^
Egg weight, g	39.6 ± 0.3^c^	49.0 ± 0.0^b^	54.0 ± 0.1^a^	55.6 ± 0.0^a^	54.9 ± 0.3^a^	56.3 ± 0.1^a^	51.2 ± 0.1^a^	52.1 ± 0.3^a^
HHEP, count	88 ± 3.4^d^	190 ± 2.8^a^	127 ± 4.2^c^	173 ± 2.1^b^	75 ± 5.6^e^	192 ± 0.7^a^	142 ± 2.1^a^	138 ± 2.5^b^
HDEP, % (week)	56.1 ± 8.3^c^ (26)	63.9 ± 3.5^b^ (26)	65.4 ± 6.5^b^ (29)	55.6 ± 7.1^c^ (41)	36.5 ± 7.8^d^ (30)	68.1 ± 5.9^a^ (35)	62.0 ± 4.6^a^ (34)	43.4 ± 3.9^b^ (32)
Feed/dozen eggs, kg	4.4 ± 2.1^b^	2.9 ± 1.3^a^	4.4 ± 1.8^b^	3.6 ± 0.9^ab^	7.6 ± 1.1^c^	2.9 ± 1.8^a^	3.5 ± 0.4^a^	4.2 ± 1.2^b^

*HHEP* hen-housed egg production, *HDEP* % lay at peak

^a-e^Means with different superscripts on the same row were significantly different at *p* < 0.05, *LSM* least square mean, *SEM* standard error of the mean

**Table 4:** Effect of location (LSM ± SEM) on age at first egg, hen-housed egg production, hen-day egg production and feed intake per dozen eggs of the breeds tested on-station

Parameters	Fulani	FUNAAB Alpha	Kuroiler	Noiler	Sasso	Shika-Brown
Fol-Hope station						
Age at first egg, d	129 ± 1.9^c^	120 ± 0.3^b^	123 ± 1.1^b^	143 ± 2.0^d^	142 ± 0.2^d^	116 ± 0.1^a^
HHEP, count	89 ± 4.3^d^	192 ± 1.8^a^	116 ± 2.3^c^	181 ± 6.2^b^	81 ± 3.6^d^	196 ± 1.0^a^
HDEP, % (week)	55.9 ± 8.5^c^ (26)	75.3 ± 6.7^a^ (26)	60.4 ± 0.8^b^ (25)	61.2 ± 7.3^b^ (34)	34.8 ± 4.2^d^ (26)	70.7 ± 0.6^a^ (35)
Feed/Dozen eggs, kg	4.2 ± 0.8^b^	2.4 ± 0.3^a^	4.1 ± 1.3^b^	4.0 ± 14^b^	6.3 ± 0.7^c^	2.6 ± 0.3 ^a^
FUNAAB station						
Age at first egg, d	122 ± 0^bc^	119 ± 1.3^ab^	117 ± 0.6^a^	116 ± 1.5^a^	123 ± 0.6^c^	122 ± 0.1^bc^
HHEP, count	86 ± 3.1^d^	187 ± 1.2^a^	138 ± 7.1^c^	162 ± 5.8^b^	67 ± 4.2^e^	188 ± 9.2^a^
HDEP, % (week)	67.9 ± 9.7^a^ (25)	54.9 ± 6.2^b^ (54.9)	47.1 ± 3.9^c^ (32)	56.7 ± 4.0^b^ (42)	26.1 ± 1.3^d^ (30)	64.9 ± 5.1^a^ (33)
Feed/Dozen eggs, kg	4.9 ± 1.1^b^	3.6 ± 0.6^a^	5.1 ± 2.1^b^	3.4 ± 0.2^a^	10.8 ± 0.4^c^	3.2 ± 2.2^a^

*HHEP* hen-housed egg production, *HDEP* % lay at peak

^a-e^Means with different superscripts on the same row were significantly different at *p* < 0.05, *LSM* least square mean, *SEM* standard error of the mean

Table 5 shows the mortality rate of the breeds. Mortality during brooding ranged from 3.6% (*Noiler*) to 33.6% (*Kuroiler*). *Noiler* (13.5%) and *Sasso* (23.7%) had higher male mortalities during growing than *Fulani* (0%), *Shika-Brown* (2.6%), *Kuroiler* (5.4%), and *FUNAAB Alpha* (7.9%), but for females, *Fulani* had the highest mortality (39.1%) while *Sasso* was lowest (1.1%). The mortality rate for *Shika-Brown* (18.0%), *Noiler* (19.2%), and *Fulani* (19.4%) was lower (*p* < 0.05) than *Sasso* (24.0%), *FUNAAB Alpha* (24.8%), and *Kuroiler* (24.5%) during laying. The effect of location on mortality rate was only significant (*p* < 0.05) during the laying phase.

**Table 5:** Mortality rates (LSM ± SEM) of the six breeds tested on-station

Phases	Breed						Station	
	Fulani	FUNAAB Alpha	Kuroiler	Noiler	Sasso	Shika Brown	Fol-Hope	FUNAAB
*Brooding*, Mortality %	5.7 ± 0.7^c^	16.9 ± 1.0^e^	33.6 ± 2.9^f^	3.6 ± 0.6^b^	9.1 ± 1.8^d^	2.0 ± 0.1^a^	13.7 ± 0.8^a^	12.6 ± 1.2^a^
*Growing*, male Mortality, %	0.0^a^	9.7 ± 1.4^d^	5.4 ± 2.6^c^	13.5 ± 2.1^e^	23.7 ± 3.6^f^	2.6 ± 0.8^b^	13.0 ± 2.9^a^	11.4 ± 1.3^a^
*Growing*, female Mortality, %	39.1 ± 0.9^d^	10.0 ± 2.5^c^	1.4 ± 0.0^a^	6.6 ± 1.8^b^	1.1 ± 0.1^a^	2.0 ± 0.1^a^	5.5 ± 1.8^a^	5.8 ± 2.3^a^
*Laying*, mortality, %	19.4 ± 1.3^a^	24.8 ± 4.4^b^	24.5 ± 2.1^b^	19.2 ± 3^a^	24.0 ± 2.2^b^	18.0 ± 1.7^a^	16.0 ± 3.2^b^	28.3 ± 2.9^a^

^a-e^Means with different superscripts on the same row were significantly different at *p* < 0.05

*LSM* least square mean, *SEM* standard error of the mean

## Discussion

The HES (70% and 68.2%) and HFE (83.2% and 83.7%) of both the foreign-sourced and locally sourced commercial hatchable eggs were similar. This suggests that apart from the genetics (Breeder flock and management), environmental factors such as proper storage and handling, general hatchery management, and appropriate incubator conditions are critical factors influencing hatchability of chicken eggs (Mauldin 2002). The HFE of all the breeds was within the range (52.4–87.0%) previously reported for both indigenous and cross-bred chickens (Alabi et al., 2012; Tadesse, 2014; Wondmeneh et al., 2011). However, only HFE of *Fulani* was lower than the range (80–90%) reported by MALDM (1993) for dual-purpose chicken breeds. The fertility and HFE of *Fulani* were low compared to the report by Adedeji et al. (2015) for *Fulani* chickens tested on-station (Fertility 85%, HFE 84%), but it was similar to that reported by Dunya et al. (2014) for Nigerian local chickens. The differences in the hatchability for *Fulani* may be associated with the variability within the *Fulani* population, since it is a local-farmer-developed strain (Olori 1992, Sonaiya 1998).

At 42 days, *Kuroiler*, *Noiler*, and *Sasso* had better growth performance than *FUNAAB Alpha* and *Shika-Brown* when compared with *Fulani.* The bodyweight gain over *Fulani* was 368%, 296%, and 245% in *Noiler*, *Kuroiler*, and *Sasso*, respectively, while it was 187% in *FUNAAB Alpha*, and 138% in *Shika-Brown*. *Noiler* was the most efficient at converting feed to live bodyweight with 50% reduction in FCR compared to *Fulani*. During the growing phase, *Kuroiler* (249%), *Sasso* (241%), and *Noiler* (211%) had higher mean live weights at 140 days for females, than *FUNAAB Alpha* (169%), *Shika-Brown* (138%), and *Fulani* (105%) when compared with the mean live weight (970 g) reported by Adedokun and Sonaiya (2002) for female Nigerian local chickens. At 140 days, the male live weights of the improved breeds were 304% (*Sasso*), 297% (Kuroiler), 267% (Noiler), 215% (*FUNAAB Alpha*), 173% (*Shika-Brown*), and 136% (*Fulani*) higher than the reported mean live weight (975 g) of male Nigerian local chickens raised on-station (Ajayi, 2010; Akinokun, 1975; Nwosu and Asuquo, 1984). This shows a cluster of fast (i.e. > 200% higher male live weights: *Sasso*, *Kuroiler*, *Noiler*, and *FUNAAB Alpha*) and slow (i.e. < 200% higher male live weights: *Shika-Brown* and *Fulani*) growing breeds, with *Sasso* and *Kuroiler* being the most efficient converters of feed to live weight.

The laying performance of *Shika-Brown*, from the cluster of slow growing breeds, was significantly superior to that of the fast-growing breeds. This is not surprising as it has been established that bodyweight is negatively correlated with laying performance (Harms et al. 1982; Oluyemi and Roberts 1979). The HHEP of *Shika-Brown*, *FUNAAB Alpha*, and *Noiler* were 226%, 224%, and 204% higher than the mean egg production of the Nigerian local chickens (85 eggs) reported by Adedokun and Sonaiya (2001), and Ajayi (2010). Also, the egg weight of *Shika-Brown*, *Noiler*, *Sasso*, and *Kuroiler* were over 140% higher than the mean egg weight (38 g) of local chickens reported by Adedokun and Sonaiya (2001) for birds reared under similar conditions. There was a 22% reduction in the mean age at first egg of the improved breeds compared with the locals (160 days), and S*hika-Brown* and *FUNAAB Alpha* were the most efficient at converting feed to eggs. According to the selection criteria (over 200% higher male live weight and egg production over local chickens) established by the ACGG project (https://africacgg.net/2016/01/13/program-meetings-set-stage-for-collaboration-and-on-farm-trials-of-improved-chicken-breeds-in-africa) for SHP-SHG, only *FUNAAB Alpha* and *Noiler* attained both the 200% higher live weight, and 200% higher egg production over the Nigerian local chickens. However, *Sasso* and *Kuroiler* achieved over 200% male live weight only, and *Shika-Brown* also achieved only over 200% egg production, compared with the local chickens.

The peak HDEP for all the breeds, except *Sasso*, *was* higher than the range (44.7–54.9%) previously reported by Adedokun and Sonaiya (2001) for Nigerian local chickens. Overall, the THI, at both Fol-Hope (30.5–30.4) and FUNAAB (30.8–31.1), was observed to plateau between the 8th and 9th months, when the peak HDEP (weeks 32–34) was recorded. This suggests that in addition to maintaining a normal (< 27.8) to moderate (27.8–28.8) THI-based stress indicator ranges, the relative stability of THI during rearing also enhances laying performance. Over 50% (10 months) of the total test period at FUNAAB were under severe to very severe (28.9– ≥ 30) THI thresholds, as against a total of 7 months (39%) at Fol-Hope. The laying phase is accounted for 90% (9 months) of the entire months with the severe to very severe THI values at FUNAAB. The birds tested in the southern Guinea savanna (Fol-Hope) had lower feed per dozen eggs (3.5), and higher HHEP (142), and HDEP (62%) compared with the dry lowland rainforest (FUNAAB). This superior laying performance could be due to the moderate THI (28.8) compared with the severe THI at FUNAAB (29.4) because egg production decreases with increasing THI values and heat stress (Kilic and Simsek 2013; Mashaly et al. 2004; Sterling et al. 2003).

Overall, the locally sourced breeds had lower mortality rates (brooding 7.3%, growing 8.2%, and laying 20.5%) than the foreign-sourced breeds (Brooding 21.3%, growing 8.7%, and laying 24.2%) which suggests that the locally sourced breeds were more adaptable to the environment than the foreign breeds. High mortality rates are reportedly associated with weakened immune systems, resulting from high levels of temperature-induced stress and inability of animals to efficiently thermoregulate (Al-Awadi et al., 1995; Furlan and Macari 2002; Mashaly et al., 2004; Mumma et al., 2006; Perreira et al., 2010). According to Ajakaiye et al. (2011) and Tao and Xin (2003), high mortality, as influenced by heat stress, also depends on the physiological state and adaptability of the breed to its prevailing environmental conditions. In addition to THI, age and bodyweight are predisposing factors to high incidence of mortality in poultry (Perreira et al. 2010). Generally, across the two stations, mortality increased from day-old to the end-of-testing, at 504 days old. In this study, there was an increased mortality rate of female birds between the growing and laying phase for all the breeds, except *Fulani*. This may be due to the heavier bodyweight of the other breeds which presents a higher metabolic activity, and the challenge of maintaining adequate thermoregulation under heat stress (Furlan and Macari 2002; Lin et al. 2006).

The low mortality observed during brooding for the locally sourced breeds, compared with the foreign breeds, suggests that the locally sourced breeds were more adaptable to the environment and could withstand the post-hatch stress and physiological changes accompanying early chick life (Yassin et al., 2009) better than the foreign breeds. Also, at the growing phase, females (6.0%) had a lower mean mortality rate than males (11.1%) which according to Leitner et al. (1989), may be associated with a generally less efficient immune response in male chickens.

The locally sourced breeds were more adapted than the foreign-sourced breeds. The breeds were differentiated into two clusters of faster-growing (*Sasso*, *Kuroiler*, *Noiler*, *FUNAAB Alpha*) and slower-growing (*Shika-Brown*, and *Fulani*) breeds. The faster-growing breeds had over 200% higher bodyweight compared with the local, unimproved chickens, while the HHEP of *Shika-Brown*, *FUNAAB Alpha*, and *Noiler* was over 200% higher than the local chickens. Based on the growth and laying performance, *Noiler* and *FUNAAB Alpha* were observed to have dual-purpose functions (i.e. for BW and egg production). On the other hand, both *Kuroiler* and *Sasso* were observed to have single-purpose function for meat (BW), while *Shika-Brown* was most suitable for egg production. The overall ranking (i.e. highest to lowest) of the breeds across the rearing phases based on growth (Final live weight and FCR) and laying performance (Age at first egg, egg weight, HHEP, HDEP and feed/dozen eggs) and survivability is *Shika-Brown*, *Noiler*, *Kuroiler*, *Sasso*, *FUNAAB Alpha*, and *Fulani*.
